# An integrated microfluidic-based biosensor using a magnetically controlled MNPs-enzyme microreactor to determine cholesterol in serum with fluorometric detection

**DOI:** 10.1007/s00604-023-05894-w

**Published:** 2023-07-19

**Authors:** Vanesa Román-Pizarro, Ángela Écija-Arenas, Juan M. Fernández-Romero

**Affiliations:** Departamento de Química Analítica, Instituto Universitario de Investigación en Química Fina Y Nanoquímica (IUNAN), Universidad de Córdoba, Campus de Rabanales, “Marie Curie” Building Annex, 14071 Córdoba, Spain

**Keywords:** Microfluidic biosensor, Electromagnet, Enzyme-magnetic nanoparticles, Optical-fiber fluorimetry, Cholesterol, Serum samples

## Abstract

This work provides a microfluidic-based biosensor to determine total cholesterol in serum based on integrating the reaction/detection zone of a microfluidic chip of a magnetically retained enzyme microreactor (MREµR) coupled with the remote fluorometric detection through a bifurcated fiber-optic bundle (BFOB) connected with a conventional spectrofluorometer. The method is based on developing the enzymatic hydrolysis and oxidation of cholesterol at microscale size using both enzymes (cholesterol esterase (ChE) and cholesterol oxidase (ChOx)) immobilized on magnetic nanoparticles (MNPs). The biocatalyst reactions were followed by monitoring the fluorescence decreasing by the naphtofluorescein (NF) oxidation in the presence of the previous H_2_O_2_ formed. This microfluidic biosensor supposes the physical integration of a minimal MREµR as a bioactive enzyme area and the focused BFOB connected with the spectrofluorometer detector. The MREµR was formed by a 1 mm length of magnetic retained 2:1 ChE-MNP/ChOx-MNP mixture. The dynamic range of the calibration graph was 0.005–10 mmol L^−1^, expressed as total cholesterol concentration with a detection limit of 1.1 µmol L^−1^ (*r*^2^ = 0.9999, *s*_*y*/*x*_ = 0.03, *n* = 10, *r* = 3). The precision expressed as the relative standard deviation (RSD%) was between 1.3 and 2.1%. The microfluidic-based biosensors showed a sampling frequency estimated at 30 h^−1^. The method was applied to determine cholesterol in serum samples with recovery values between 94.8 and 102%. The results of the cholesterol determination in serum were also tested by correlation with those obtained using the other two previous methods.

**Graphical abstract:**

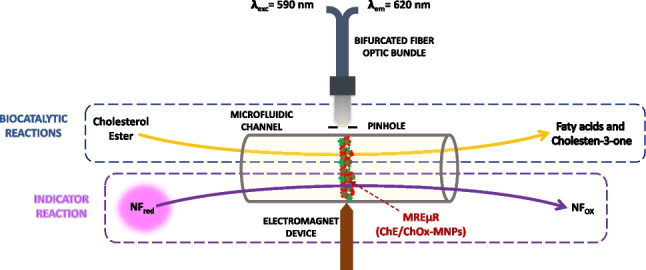

**Supplementary information:**

The online version contains supplementary material available at 10.1007/s00604-023-05894-w.

## Introduction

Cholesterol is an essential lipid that acts as a structural constituent of plasma membranes and is a precursor to steroid hormones, bile acids, vitamin D, and other vital molecules. The serum cholesterol level is an indicator in diagnosing and preventing various cardiovascular diseases, hypothyroidism, nephrotic syndrome, diabetes, and liver diseases. Its synthesis in the body is directly regulated by its blood levels. Higher intake causes a net decrease in endogenous production, while lower intake has the opposite effect. Therefore, its clinical control is significant in clinical laboratories [1].

Currently, the main methods for determining cholesterol are based on enzymatic reactions. In addition, several methods and techniques have been developed to determine cholesterol based on chromatographic, capillary electrophoresis, electrochemical, photometry, and fluorimetry techniques [2–8]. These methods are sometimes time consuming and non-specific processes, using high volumes of samples and reagents to be carried out. However, these methods could be easily automated and miniaturized, showing enough sensitivity, selectivity, simplicity, and faster responses. There are recent reviews published that describe several types of cholesterol biosensors (based on enzymatic or non-enzymatic principles) coupled with several physicochemical transductors (electrochemical, optical, piezoelectric, between others) that could be applied to determine cholesterol in fruits, beverages, and clinical samples [9–12]. The immobilization of enzymes through magnetic nanomaterials provides an excellent way for their temporary retention in the active microzone of the biosensor, providing stability, easy regeneration, and a considerable reduction in costs without altering the properties of the biosensor. Bioactive materials based on magnetic retention also offer a handy tool for developing miniaturized flow biosensors. Introducing enzyme-nanomaterial complexes in dynamic systems operating at the nano/micrometer scale can also improve the excellent changes in physical, chemical, and electronic properties due to the scale change.

Microfluidic devices have been shown to have great potential in biological applications, with numerous advantages, including low sample/reagent consumption, high sample throughput, and analytical process integration capabilities [13]. In 2010, Wisitsoraat et al. reported a novel cholesterol detection system using a functionalized carbon nanotube (CNT) electrode in a poly(dimethyl)siloxane/glass-based flow injection microfluidic chip. [14]. This electrochemical flow-injection microfluidic system could probably be the first attempt to develop a flow biosensor with the enzyme ChOx immobilized in CNT and manufactured in poly(dimethyl)siloxane.

Integrating active sensor zones at the microscale based on immobilizing the biomaterial is one of the most important tasks to expand the applicability of microfluidic-based biosensors. The integration of optical physicochemical transducers into the microfluidic chip implies added complications. Immobilizing the biomaterials in the microchannels is critical for the proper biocatalytic microreactor functionality [15]. These materials can be immobilized on the microfluidic channel walls or by immobilization on support forming a microreactor within the microchannel. These immobilized enzymatic microreactors can be prepared differently (entrapment in nanoporous scaffolds, chemical bonding with controlled pore glass (CPG), or covalent bonding with magnetic nanoparticles (MNPs)). Using MNPs as an enzyme support material is an excellent alternative to constructing an enzyme microreactor.

This research presents an integrated microfluidic-based biosensors that incorporate at the same reaction/detection zone the co-immobilized ChE/ChOx-MNPs as microreactor (MREµR) magnetically retained joined with the physicochemical transduced using a bifurcated fiber optic bundler (BFOB) connected to a conventional spectrofluorometer and behaving as a microfluidic scale biosensor where the active microzone and the physicochemical transducer are integrated.

## Material and methods

Materials, apparatus, instruments, and the synthesis of magnetic nanoparticles and covalent immobilization of the enzymes are provided in the Electronic Supplementary Material (ESM).

### Enzymatic and indicative reactions

**Fig. 1:**
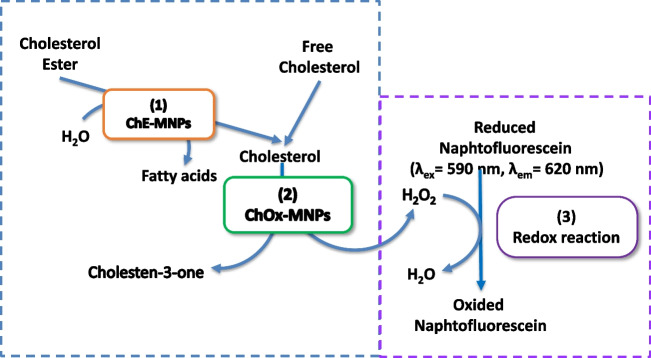
Schematic representation of the enzyme and redox reactions involved in the biosensor structure and its operating principle

The analytical system chosen involves three sequential reactions at the reaction/detection zone of the integrated microfluidic-based biosensors, depicted in Fig. 1. The two first enzymatic reactions occurred at the bioactive microzone that provides the subsequent hydrolysis of the esterified cholesterol using the cholesterol esterase (ChE) and the oxidation of free cholesterol in the presence of the cholesterol oxidase (ChOx), giving the corresponding ketone and hydrogen peroxide. Both enzymes were immobilized separately on MNPs and mixed and constituted the active microzone of the biosensor. The hydrogen peroxide formed is monitored by a third redox reaction consisting of naphtofluorescein (NFred) oxidation that exhibits native fluorescence at the excitation and emission wavelengths of 590 and 620 nm, respectively. As a result of this redox reaction, a fluorescence decay was achieved in the physicochemical transduced that provides the final net signal directly related to the total cholesterol concentration in the samples.

### The integrated microfluidic-based biosensor

**Fig. 2:**
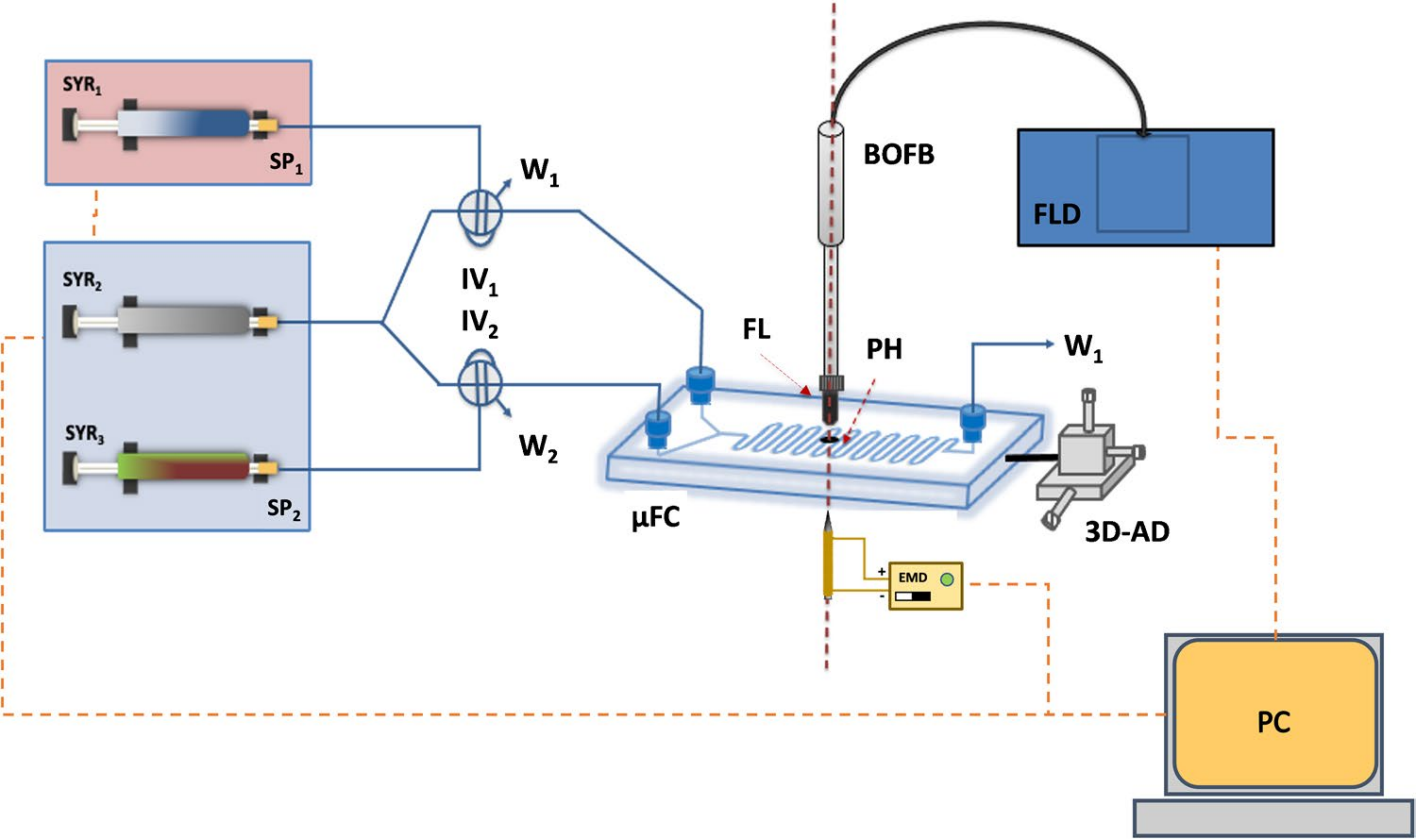
Manifold to determine total cholesterol in serum using the integrated microfluidic-based biosensors. SP_1_ and SP_2_, pneumatic drive pumps; SYR_1_, SYR_2_, and SYR_3_, push syringes; IV_1_ and IV_2_, injection valves; w_1_, w_2_, and w_3_, fluid wastes, µFC, microfluidic device; BOFB, bifurcated fiber optic bundle; F.L., focussed microscope lens; PH, pinhole; 3D-AD, *x*–*y*-*z* alignment device; FLD, spectrofluorimetric detector; EMD, “lab-built” electromagnetic device; PC, computer control device. The orange lines represent the control points of the computer system. The red dotted line represents the alignment of the optical system with the reaction/detection zone. The chip holder is not included in the scheme. Also, some of the devices are not presented in their accurate scale

A scheme of the integrated microfluidic-based biosensor system that incorporates the microfluidic with the optical luminescence and the electromagnet devices is shown in Fig. 2. It comprises the fluidic propel pumps, the injection valves, and the microfluidic biosensor platform, including the chip holder containing the microfluidic chip, the “lab-built” electromagnet device, and the optical system assembly.

**Fig. 3:**
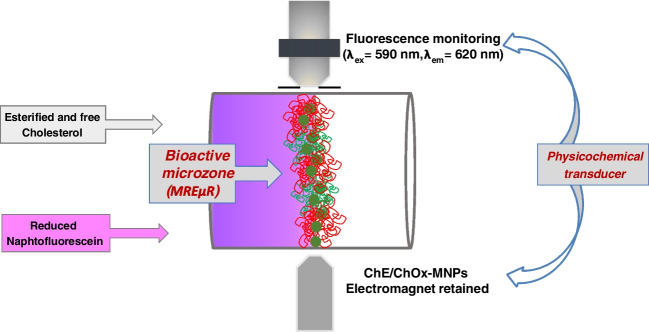
Detail of the biosensor integrated into the reaction/detection zone formed by its two parts (bioactive microzone and physicochemical transducer)

Figure 3 shows a detailed diagram of the parts that make up the integrated on-flow biosensor (active microzone and physicochemical transducer) included in the reaction/detection zone of the microfluidic chip.

The optical system was a block integrated by the bifurcated optical fiber bundle (BFOB) focused through a microscope lens partially covered with a 1-mm-diameter pinhole (PH) located at a focused distance of 5 mm to the microfluidic channel. This optical device brings the light from the excitation source to the microfluidic platform and collects the emitted light to the spectrofluorometer FluoroMax-4P. The excitation and emission wavelengths were performed at 590 and 620 nm, respectively, corresponding with the maximum excitation and emission wavelengths of the reduced naphtofluorescein indicator. The excitation beam was carried out by the first fiber bundle of the bifurcate optical fiber and focused directly through the pinhole-microscope objective to the microchannel glass microreactor, covering the optical zone where the activate electromagnet device retained the MREµR (approx. between 1 and 2 mm length size). When an injected plug containing the reduced NF and the sample solution containing cholesterol passed through the bioactive zone, the emission beams originated by the excess of reduced NF not consumed in the enzymatic/redox reactions are collected by the second fiber optic bundle that is carried out through the returned beam to the spectrofluorometer detector. The ESM displays photographic images of the integrated microfluidics-based biosensor and some auxiliary devices. Figure S1 incorporates both the biocatalytic/redox reaction formed by the MREµR in which are magnetically retained ChE/ChOx-MNP active microzone and the physicochemical transduction zones formed by the electromagnet device and the bifurcated optical fiber bundle (BOFB). The extension of the redox reaction was transduced as the signal response, translated through the optical device to the conventional spectrofluorometer.

### Synthesis of magnetic nanomaterial and immobilization of the enzymes

The synthesis of the nanomaterial and their characterization are presented in the ESM. Figures S2 and S3 show the nanomaterial characterization by TEM, SEM–EDX, XPS, and DLS. Furthermore, the immobilization efficiency of the enzymes in the MNPs is also shown.

### Determination of cholesterol concentration

In the first step, as required, both pneumatic drive pumps propel the solution in the three push syringes (SYR1, SYR2, and SYR3). Then, the suspension containing a mixture of ChE/ChOx-MNPs prepared at a 2:1 ratio in phosphate buffer solution (0.05 M, pH 7.4) was injected through the injection valve IV_1_ at a flow rate of 25 µL min^−1^. The ChE/ChOx-MNP plug was retained, forming the MREµR at the exact microfluidic chip-focused line provided by the activated electromagnet device. This step consumes a retention time of Δ*t*_1_ = 300 s, which is available for a complete working session. In a second step, the solution containing SDS (4 mmol L^−1^), NF (0.5 mmol L^−1^), and cholesterol (as standard or as diluted human serum) was then injected using the second injection valve IV_2_. The enzyme reaction occurred through the reaction/detection zone, reducing the NF, which was monitored at a wavelength of *λ*_ex_ 590 nm and *λ*_em_ 620 nm, using a 5/5 slit ratio and a detector gain of 950 V. Adding the anionic surfactant SDS to the injected solution provides an additional luminescence signal enhancement, characteristic of many long-wavelength fluorophores such as NF [16]. The analytical signals were obtained using the microfluidic-based biosensor that provides the parameter to establish the net analytical signal and a release time (Δ*t*_2_), which was enough to reach the baseline signal. The net signal was calculated as the differences between the peak areas obtained in the absence (*I*_0_) and the presence (*I*_i_) of the analyte monitored for 120 s. Also, the release time was used to estimate the sampling frequency of the microfluidic biosensor response. Each standard or sample solution was injected in triplicate.

### Analysis of serum samples

Several human serum samples were processed to determine the cholesterol concentration. The samples were adequately diluted using 0.05 mmol L^−1^ phosphate buffer solution (pH 7) and analyzed as described above.

### Comparison methods

The results obtained by applying the microfluidic-based biosensor to the analysis of human serum samples were compared with those obtained using two previous methods based on the clinical autoanalyzer (ILab-600) and a flow-injection system developed by the authors [17]. The methods were compared using a similar commercially available cholesterol standard as a reference. Calibrations were carried out using material traceable to the cholesterol lipid standardization laboratory at the Centers for Disease Control. Serum samples were assayed in parallel with the three assayed methods, and the least-squares regression methodology was used to compare the results. Two serum standard control at low (2.7 ± 0.35 mmol L^−1^) and high (5.2 ± 0.25 mmol L^−1^) cholesterol concentration levels should be run as unknown samples to the quality control. All experiments were replicated three times. The method’s applicability was also tested using the paired t-test by comparing the results obtained to analyze fifty human serum samples using the new microfluidic-based biosensor and the previously reported methods (ILab-600 and FI-method).

## Results and discussion

### Study of the experimental variables of the method

The variables affecting the synthesis of ChE/ChOx-MNPs involved in the method for cholesterol determination were studied using a similar procedure developed previously [17] and presented in the ESM.

Concerning the variables affecting the enzymatic and redox reactions at microfluidic-based biosensors, the method was developed using phosphate buffer solution as the carrier to transport, first, the ChE/ChOx-MNPs and, second, to transport the cholesterol, NF, and SDS mixture. Hydrodynamic, instrumental, and chemical variables were optimized, as shown in Table 1, including the ranges assayed and the values chosen for each variable. All variables were studied in triplicate.

**Table 1:** Study of the variables involved in the microfluidic-based biosensor

Type of variables	Variable	Range studied	Optimal value
Hydrodynamic	Flow rate, µL min^−1^	10–100	50
	Injection volume IV_1_, µL	50–250	100
	Injection volume IV_2_, µL	1–5	5
	Measurement time, s	100–500	120
Instrumental	*λ*_ex_, nm	200–800	590
	*λ*_em_, nm	200–800	620
	Excitation slit, nm	1–10	5
	Emission slit, nm	1–10	5
	Detector gain, V	650–950	950
	Focalized lens distance, mm	1–10	5
	Pinhole distance, mm	1–3	1.5
	Electromagnet power, V	-	6
Chemical	pH	6.5–8	7
	[Phosphate], mmol L^−1^	10–100	50
	SDS, mM	0–10	4
	Naphtofluorescein, µmol L^−1^	100–1000	500
Physical	Temperature, °C	-	30

**Fig. 4:**
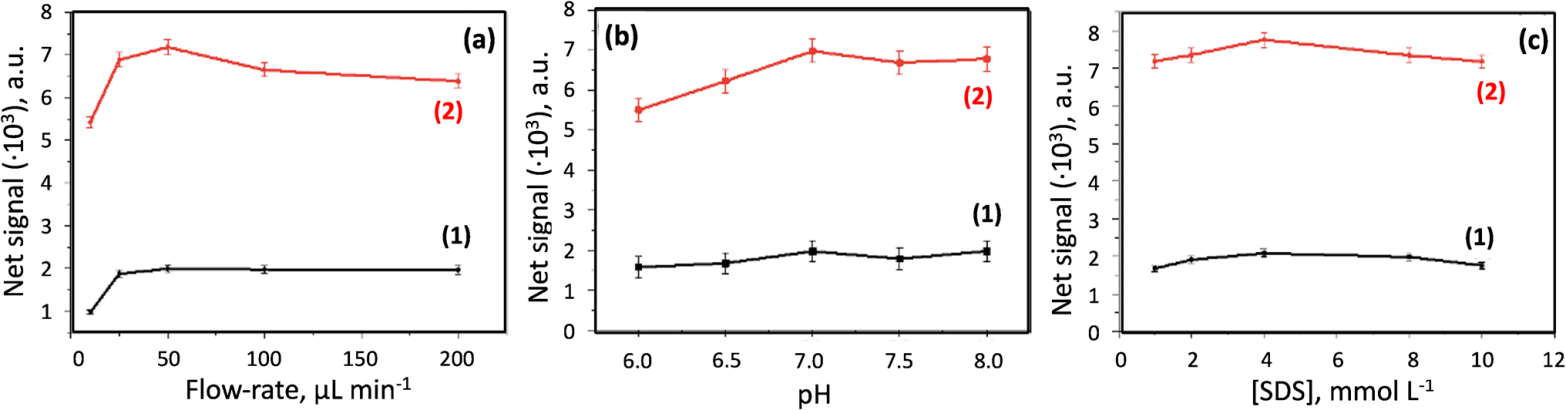
Influence of some experimental variables on the flow system: **a** flow rate for the development of biocatalytic and redox reaction, **b** pH, and **c** naphtofluorescein concentration. [Na_2_HPO_4_] = 50 mmol L^−1^, [SDS] = 4 mmol L^−1^, [cholesterol] = 0.25 mmol L^−1^ (1), and 1 mmol L^−^.^1^ (2)

All the assays were carried out at a fixed temperature of 30 °C to avoid degradation of the enzymes. This was achieved by immersing the tubes with the solutions in a thermostat bath and also controlling the room temperature. The injection volumes chosen to introduce ChE/ChOx-MNPs (IV_1_) and cholesterol (IV_2_) solutions into the system were 100 µL and 5 μL, respectively, using an adequate flow rate of 50 µL min^−1^ (see Fig. 4a). The influence of pH was studied in a range between 6 and 8; as shown in Fig. 4b, a pH of 7 was chosen as optimal. Figure 4c shows the influence of SDS concentration on enhancing the analytical signal, in which a 4 mmol L^−1^ concentration is suitable to obtain the maximum net signal in all instances.

### Features of the microfluidic-based biosensor

The calibration graph of the method was obtained under the optimum experimental conditions and using the difference in the peak area obtained in the absence and the presence of the analyte, measured at *λ*_ex_ = 590 nm and *λ*_em_ = 620 nm, the analytical parameter (*y* = net signal). Table S1 in the ESM shows the analytical characteristics of the proposed method showing the parameters of the equation that defines the methodological calibration curve, the LOD, and the dynamic linear range, as well as the accuracy of the method calculated as a percentage of relative standard deviation in the minimum and maximum error zones of the curve and testing two serum control samples (normal and pathologic levels).

**Fig. 5:**
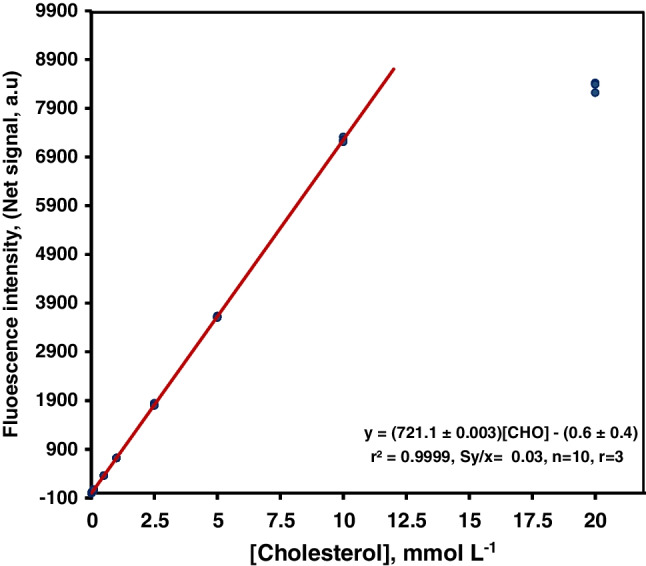
Calibration graphs obtained using the microfluidic-based biosensor

The calibration graph equation was *y* = − 0.6 (± 0.4) + 721.1 (± 0.003) · [CHO] being the cholesterol concentration expressed as mmol L^−1^, and the regression coefficient was 0.9999, with a residual standard error of 0.03 (*n* = 10, *r* = 3). The linear range of the calibration graph was 0.005–10 mmol L^−1^ cholesterol, and the limit of detection (LOD), calculated according to IUPAC recommendations [18], was 1.1 µmol L^−1^. Furthermore, Fig. 5 shows the calibration graph obtained. The precision of the method, expressed as a percentage of relative standard deviation (RSD%), was studied at two concentration levels corresponding to the calibration graphs’ maximum and minimum error zones and the certified values established for the two serum control samples used. The precision was established at the concentration of 0.1 and 5 mmol L^-1^, obtaining RSD% values of 2.1 and 1.3 %, respectively. Also. 2.7 and 5.2 mmol L^−1^ were achieved for the certified values of normal and pathologic levels, respectively. The selectivity was studied by assaying four potential interference compounds: ascorbic acid, uric acid, glucose, and lactic acid. The results revealed that these compounds did not cause interference at least 100 times the interferent/analyte ratio. The estimated sampling frequency under the working conditions was about 30 h^−1^.

A comparison of the proposed method features with other previously reported methods for cholesterol determination is shown in Table 2. Few methods have a lower detection limit than the reported method, but it is compensated by the extensive linear range in which it can work. The immobilization described here is a procedure more complex than others presented, in which the enzyme is immobilized with only electrostatic interactions. However, the new method presents one of the bigger stabilities, maintaining 90% of the enzyme activity for over 2 months, caused by this type of immobilization using the carbodiimide reaction. The measurement time is similar to the needed for microfluidic devices and inferior to the necessary for conventional methods.

**Table 2:** Comparison of the analytical features for cholesterol determination with different methods

Method	Enzyme	Type of immobilization	Support	LOD (µmol L^−1^)	Linear range (µmol L^−1^)	Measurement time (min)	Stability	Sample	Reference
Fluorescence in conventional flow	ChE/COx	Covalent	MNPs	650	1.55·10^3^–1·10^5^	3	90% after 2 months	Serum	17
Amperometry	ChE/COx	Electrophoretic deposition	Cu_2_ONPs-chitosan	27	40–1.22·10^3^	0.05	82% after 2 months	Serum	19
Amperometry	COx	Adsorption	AuNPs-MWCNTs	2.2	10–1.05·10^3^	30.4	78% after 8 days	Serum	20
Amperometry in a paper-based system	COx	Non-immobilized	Nanoporous gold in screen-printed electrode	8.36	50–6·10^3^	10	-	Serum	21
Fluorescence	COx	Non-immobilized	MnO_2_ NS-FAM	0.33	1–300	40	-	Serum	22
Fluorescence	COx	Electrostatic interaction	ZnO NWs	0.24	0.24–1.1	120	-	Serum	23
Amperometry in a microfluidic system	COx	Covalent	CNTs	-	1.29·10^3^–4·10^6^	1	80% after 1 month	Serum	24
Amperometry in a microfluidic system	COx	Electrostatic interaction	NiO film	100	120–1.02·10^4^	1	-	Serum	25
Fluorescence in a microfluidic system	ChE/COx	Covalent	MNPs	1.1	5–1·10^4^	2	90% after 2 months	Serum	This work

### Application of the method

The applicability of the microfluidic biosensor for cholesterol determination in serum samples has been performed by analyzing twenty serum samples of humans. All samples have been diluted 100 times in buffer solution, and the results obtained are shown in Table 3. As shown in this table, the cholesterol concentration in samples provides an acceptable correlation between the microfluidic-based biosensor and the two methods. A recovery study has also been conducted to typify possible interferences by using the standard addition method by fortifying twenty serum samples with two cholesterol concentration levels (0.25 and 5 mmol L^−1^). They obtained recovery values between 94.8 and 102%.

**Table 3:** Application of the method

Sample	Conventional method (mmol L^−1^)	FI method (mmol L^−1^) ^a^	Microfluidic-based biosensor (mmol L^−1^) (this work)	Recovery %^b^	
				1ª add	2ª add
1	6.20	6.30	6.40	99.5	95.0
2	6.30	6.10	6.50	99.2	95.0
3	5.90	5.90	5.80	101.2	98.0
4	4.40	4.50	4.20	101.0	99.0
5	6.40	6.30	6.30	98.3	101.0
6	5.20	5.60	5.60	100.0	102.0
7	8.60	8.20	8.70	96.5	100.4
8	5.50	4.90	5.80	99.0	101.7
9	7.20	7.30	7.80	98.0	96.0
10	6.80	6.60	7.10	102.4	99.0
11	6.90	6.50	5.90	94.8	95.9
12	5.50	4.90	4.90	96.0	99.0
13	8.50	7.90	8.30	101.4	100.0
14	7.70	8.20	7.90	100.3	101.3
15	6.50	6.90	6.40	95.0	96.0
16	4.90	4.80	4.60	99.0	95.0
17	5.20	5.20	5.00	99.4	99.0
18	8.80	8.40	7.90	102.2	97.8
19	9.20	8.80	9.00	98.0	100.3
20	6.80	5.90	6.50	96.0	98.8

^a)^ Developed according to the FI method features [17]. ^b)^ First addition 0.25 mmol L^−1^ and second addition 5 mmol L^−1^. This value represents the concentration average of each concentration obtained in triplicate

The comparison of these results has also been validated by applying the three methods on fifty serum samples and studying the paired data test-t at a significance level of 95%, finding no significant differences between results provided by both methods. Additional information for comparing comparison and developed methods is provided in Fig. S5 in the ESM. No significant differences between results provided by methods can be found in this figure.

## Conclusions

The applicability of biosensors based on the integration of microfluidic systems to carry out the automatic determination of cholesterol based on the use of enzymes immobilized by magnetic nanoparticles in a microfluidic system has been demonstrated. The research involves the adaptation of the biosensor microzone by using two magnetically retained ChE/ChOx enzymes in the reaction zone integrated with detection. The detection is carried out by incorporating a fiber optic bundled focused on the microfluidic device and transferring the instrumental signal to a conventional spectrofluorometer. During preconcentration in the reaction/detection zone, enzymes using the electromagnetic device achieve a very low LOD, while the long-wavelength fluorophore NF and surfactant SDS are used as positive reactant to obtain good selectivity and increase sensitivity. As can be seen, the LOD of the microfluidic method (1.1 μmol L^−1^ is lower than that obtained using the method previously developed by the authors (which has a LOD of 0.65 mmol L^−1^). The developed method has a LOD like that previously reported in the literature, but a larger linear range and a lower measurement time than those that do not use microfluidic systems. The use of the enzyme immobilized on the MNPs, and its preconcentration in the detector, instead of directly introducing the reagent into the microfluidic, with the consequent high consumption of reagent, improves the analytical signal and decreases the LOD. The method has been successfully applied to the analysis of different human blood samples obtaining better results than those obtained using the ILab-600 autoanalyzer and the FI method developed previously.

## Supplementary information

Below is the link to the electronic supplementary material.

Supplementary file1 (DOCX 926 KB)

## Data Availability

The authors confirm that the data supporting the findings of this study are available within the article and its Supplementary Electronic Material.
