# Supplementary material for: An integrated microfluidic-based biosensor using a magnetically controlled MNPs-enzyme microreactor to determine cholesterol in serum with fluorometric detection
Source: Mikrochim Acta. 2023 Jul 19;190(8):303. doi: 10.1007/s00604-023-05894-w (PMC10354181; doi:10.1007/s00604-023-05894-w)
Supplement: Supplementary file 1 — Supplementary file1 (DOCX 926 KB) [file 604_2023_5894_MOESM1_ESM.docx]

**Electronic Supporting Material on the Microchemical Acta publication:**

**TITLE:** An integrated microfluidic-based biosensor using a magnetically controlled MNPs-enzymes microreactor for the determination of cholesterol in serum with fluorometric detection

**AUTHORS:** Vanesa Román-Pizarro, Ángela Écija Arenas, Juan M. Fernández-Romero*

Departamento de Química Analítica, Instituto Universitario de Investigación en Química Fina y Nanoquímica (IUNAN), Universidad de Córdoba, Campus de Rabanales, “Marie Curie” Building Annex, E-14071 Córdoba, España

* Corresponding author: Prof. Juan-Manuel Fernández-Romero

ORCID: 0000-0001-8443-1358

E-mail address: qa1feroj@uco.es

First author: Vanesa Román Pizarro

ORCID: 0000-0002-1116-0427

E-mail address: q52ropiv@uco.es

Middle autor: Ángela Écija Arenas

ORCID: 0000-0003-3240-5769

E-mail address: q92ecara@uco.es

**Materials and methods**

**Materials**

Cholesterol (CH), hexadecyltrimethylammonium bromide (CTAB), naphtofluorescein (NF), hydrogen peroxide, N, N′-dicyclohexylcarbodiimide, sodium dodecyl sulfate (SDS), Triton X-100, sodium hydroxide, and tris(hydroxymethyl) aminomethane (Tris), N, N'-dimethylformamide, cholesterol esterase (EC 3.1.1.13, cholesterol esterase from Pseudomonas sp., ChE), cholesterol oxidase (EC 1.1.3.6, cholesterol oxidase from microorganism, COx) were purchased from Sigma (Sigma-Aldrich, Steinheim, Germany, http://www.sigmaaldrich.com). Iron (II) chloride, hydrochloride acid, sodium phosphate, and other common reagents were also purchased from Merck (Merck, Darmstadt, Germany, http://www.merck.com), and iron (III) chloride from Panreac (Panreac Química, Barcelona, Spain, http://www.panreac.es/es). Phosphate buffer solutions (50 mM pH 7) were prepared using deionized water purified with a Milli-Q system (Millipore, Bedford, MA, USA, http://www.emdmillipore.com). Since cholesterol does not dissolve well in standard buffer solutions, Cholesterol stock solutions (1 and 15 mM) were prepared in phosphate buffer 50 mM with 5% (v/v) chloroform.

**Apparatus and Instruments**

**Fig. S1.** displays photographic images of the integrated microfluidics-based biosensor and some auxiliary devices. A glass microreactor model FC_R150.332.2 with 12 x 24 mm dimensions and an internal volume of 6 μL was assembled to a fluidic chip-holder (R4515) (Micronit, The Netherlands, www.micronit.com). The fluorescence emission was collected using an FL-3000/FM4-3000 optical fiber bundler assembled to a Horiba Scientific Fluoromax-4P spectrofluorometer (Horiba Scientific, France, www.horiba.com/scientific/). This instrument was equipped with a 150 W Xenon lamp that operates at 50-60 Hz, with a sampling rate between 1 ms to 160 s with a spectral resolution lower than that 0.5 s. It is also provided by a photomultiplier tube that operates between 200 to 850 nm. It has an acquired frequency until 4·105 count s-1, a half lifetime between 200 ps to 0.1 s, and a resolution time less than 7 ps. The FluorEssence Software (HoribaScientific) and OriginPro 2018 64-bit Software (OriginLab Co. 2018, Northampton, MA) were used for spectrofluorometer control and signal acquisition. The optical fiber bundler and the microchip were adapted to an X-Y-Z positioner (Oriel Instruments, USA, www.newport.com/oriel/) to allow fine positioning adjustments. The flow was driven through the microfluidic reactor using two single syringe pumps NE300 Micronit microfluidics (Micronit, The Netherlands, www.micronit.com) where 5 mL syringes (Terumo, Madrid, España; www.terumo.es) were mounted. Two Cheminert VA-CN2 injection valves (Valco, Teknokroma, Barcelona, Spain, www.teknokroma.es) were used to inject the enzymes-MNPs mixture and the sample and reagents solution. The resulting flow was connected to the microfluidic glass chip using polytetrafluoroethylene (PTFE) tubes (i.d. 0.25 mm) and the adequate PEEK connector.


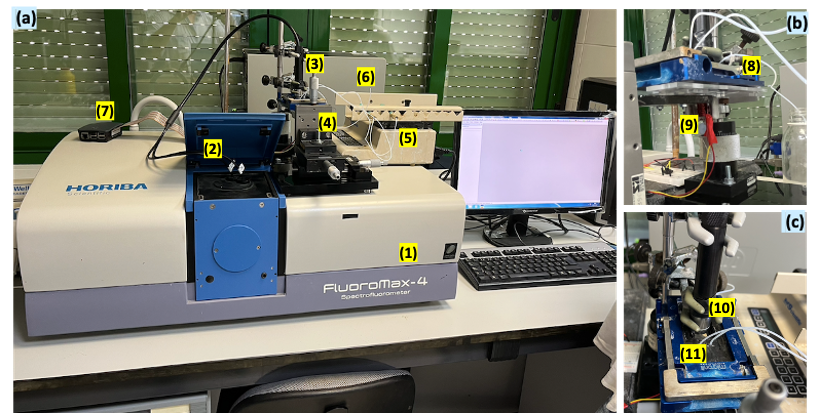


**Fig. S1.** Photographic images of the integrated microfluidics-based biosensor. **a** depict the integrate system which includes, (1) the conventional spectrofluorimeter, (2) the device which connected the optical fiber bundler-BOFB- (3) to the sample compartment detector, (4) X-Y-Z alignment device, (5) the pneumatic drive pumps, which included the push syringes and the IV_1_ and IV_2_, injection valves, (6) and (7) the PC computer and the raspberry-pi control system; **b**, detail of the microfluidic chip (8) and the “lab-built” electromagnet device (9); and **c**, detail of the focussed optical fibre bundler (10) and the microfluidic chip (11)

A desktop laboratory MPW-350R centrifuge (MPW Med. Instrument, Warsaw, Poland) with a cooling chamber rotating and an angle rotor HSL-11199 (45º, 12 x12 x1.5 mL, max. speed= 18000 rpm (RCF= 24088 g and r_min_/r_max_ of 3.5/6.25) was used for the NMs separation. Transmission electron microscopy (TEM) images for characterization of the NMs were obtained using a CM-10 Philips microscope (Philips Research, Eindhoven, The Netherlands; http://www.research. philips.com) with 0.5 Å~ 0.34 nm resolution and equipped with a digital mega view III camera. Copper grids (200C-FC) coated with a Formvar carbon film 200 mesh supplied by Aname (Madrid, Spain; http://www.aname.es) supported TEM experiments. A VorTemp 56 LA-S2056 shaking incubator (Labnet Int. Woodbridge, USA) and an ultrasound bath were used. Zeta potential measurements were carried out with a disposable polystyrene cuvette filled with 1 mL of the suspension and analyzed with the particle size in the backscattering mode at an angle of 173° (Malvern Zetasizer Nano ZSP ZEN6000 analyzer, Malvern Instruments, Spring Lane South, Worcestershire, UK, www.malvernpanalytical.com) at 25 °C after an equilibration time of 120 s.

Other apparatus, such as a conventional oven, an ultrasound bath, and an MPW-350R centrifuge (MPW Med. Instrument, Warsaw, Poland, www.mpw.pl) with a cooling chamber rotating, equipped with an angle rotor HSL-11199 (45º, 12×12×1.5 mL, max. speed = 18000 rpm, 24088×g RCF and r_min/max_ = 3.5/6.25) were used for the enzymes-MNPs synthesis.

**Synthesis of magnetic nanoparticles and covalent immobilization of the enzymes**

The MNPs were prepared following the previously described co-precipitation method [1] Briefly, 25 mL of an aqueous solution containing 0.4 mol L^-1^ iron (III) chloride, 0.4 mol L^-1^ iron (II) chloride, and 40 mM hydrochloric acid was dropwise added to 250 mL of 1.5 mol L^-1^ sodium hydroxide solution under vigorous stirring with a Vortex. Black brown MNPs were immediately formed, separated using a commercial neodymium magnet (NdFeB), and washed 3 times with deionized water. The Fe_3_O_4_NPs were then collected by centrifugation at 5352.80 g for 15 min and dispersed again in 100 mL of deionized water. The synthesized MNPs were avoided because they were characterized by the same research group [2-6].

The enzyme immobilization on the MNPs surface is a critical step in the bioactive zone of the microfluidic biosensor. The immobilization of each enzyme (ChE and COx) was developed separately using a previous method based on the covalent crosslinking reaction of the EDC with the recently synthesized MNPs [7]. Briefly, 50 –70 mg of MNPs was added to 1 mL of phosphate buffer (0.05 mol L^-1^, pH 7.4). The mixture was sonicated for 15 min after adding 0.5 mL of EDC solution (0.02 g/mL in phosphate buffer (0.05 M. pH 7.4). Following the EDC activation, 2 mL of each enzyme selected (0.25 mg mL^-1^) was added, and the reaction mixture was sonicated for 30 min at 4 °C in a sonication bath. The mixture was centrifuged at 1777 g. The precipitates containing MNPs and (enzyme-MNPs) were washed with phosphate buffer pH 7.4 and 0.1 mol L^-1^ Tris, pH 8.0, 0.1 mol L^-1^ NaCl, and then used for activity and stability measurements. Each solution containing immobilized enzyme-MNPs is preserved at 4 ºC in phosphate buffer.

**Characterization of the hybrid ChE/COx-MNPs**

The synthesized materials were characterized with different techniques to obtain information about MNPs and ChE/COx--MNPs characterization was summarized in this section because similar enzymes-MNPs complexes have been widely characterized by different techniques and were discussed in previous research [5, 7]. **Fig. S2**, shows the TEM characterization of MNPs **a** and ChE/COx-MNPs, **b** showed that they have spherical structures with an average diameter of 15 ± 2 nm. **c** and **d**, depict the morphology and elemental composition of these MNPs which were studied by X-ray photoelectron spectroscopy (XPS) and scanning electron microscopy, equipped with an energy dispersive detector (SEM-EDX). The average hydrodynamic diameter of MNPs was obtained by dynamic light scattering (DLS) at 10 nm with a polydispersity of 0.10.


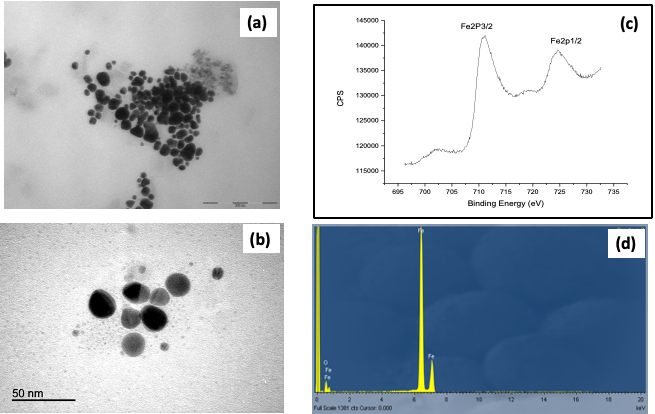


**Fig. S2.** Characterization of the MNPs used to immobilize both ChE and ChOx enzymes. **(a, b)** TEM images of the MNPs and ChE/ChOx-MNPs, respectively. **(c-d)** Morphology and elemental composition of these MNPs studied by X-ray photoelectron spectroscopy (XPS) and scanning electron microscopy, equipped with an energy dispersive detector (SEM-EDX)

The number and size average distribution of the synthesized MNPs is shown in **Fig. S3**, where approximately 95% of the MNPs appear to be 9.5 - 10.5 nm.

**
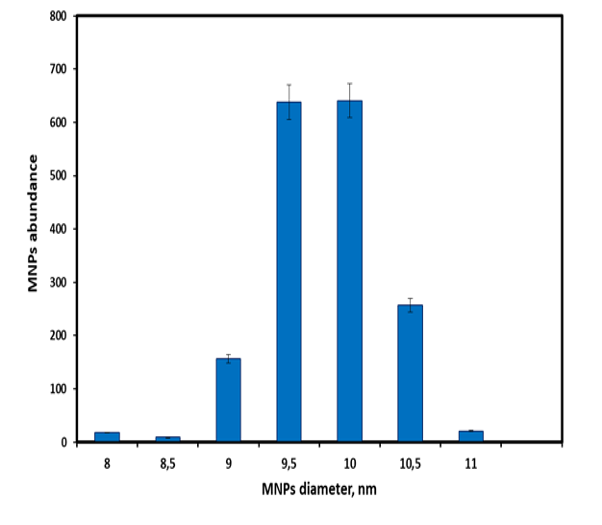
**

**Fig. S3.** The standard average number and size distribution of the synthesized MNPs, where approximately 95% of the MNPs appear to be 9.5 -10.5 nm.

The immobilization efficiency was tested using the conventional enzymatic reaction with the enzyme remaining in the solution before and after their immobilization. In both cases using ChE and COx separately, the percent of efficiency was about 98.2 ± 0.5 %.

**Features of the microfluidic-based biosensor**

**Table S1.** Analytical features of the method

|  | | **Microfluidic-based biosensor** |
| --- | --- | --- |
| Equation parameters | *Intercept Slope*  *r^2^* | -0.6 (± 0.4)  721 (± 0.003)  0.9999 |
| LOD (μmol L^-1^)  Lineal range (mmol L^-1^) | | 1.1  0.005 – 10 |
| RSD% ^a^  Max. error  Min. Error | | 2.1  1.3 |
| Serum control ^b^  Level 1  Level 2 | | 2.7  5.2 |
| ^a)^ RSD% values achieved for the microfluidic biosensor at 0.1 (max. error) and 5 mmol L^-1^ (min. error), and ^b)^ Control serum level 1 (2.7 mmol L^-1^) and level 2 (5.2 mmol L^-1^). | | |

**Application of the method**

The comparison of these results testing fifty serum samples using the microfluidic-based biosensor in comparison with both reference methods has been performed using the paired data test-t at a significance level of 95%, finding no significant differences between results provided by both methods. **Figure S4a-b**, depict the correlation achieved in all instances.

**
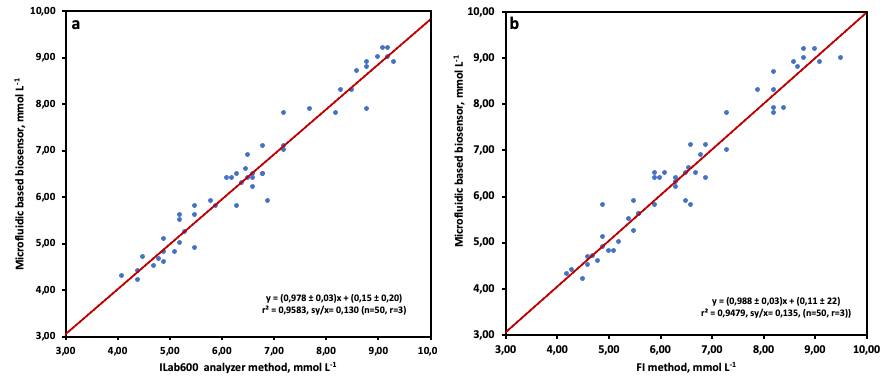
**

**Fig. S4.** Correlation between the microfluidic-based biosensors and the reference method: **a** the ILab600 analyzer method and **b** the FIA method

**References**

1. Cui YR, Hong C, Zhou YL, Li Y, Gao XM, Zhang XX. (2011) Synthesis of oriented bioconjugated core/shell Fe3O4@Au magnetic nanoparticles for cell separation. Talanta. 85: 1246–1252. doi:10.1016/j.talanta.2011.05.010.
2. Román-Pizarro V, Gómez-Hens A. Fernández-Romero JM. (2018) Applicability of Fluorescent Hybrid Magnetoliposomes for the Determination of Reactive Oxygen Compounds in Food. Food Anal. Methods. 11: 2376–2383. https://doi.org/10.1007/s12161-018-1220-3
3. Román-Pizarro V, Fernández-Romero JM, Gómez-Hens A. (2017) Automatic determination of coenzyme Q10 in food using cresyl violet encapsulated into magnetoliposomes. Food Chem. 221 (2017) 864–870. https://doi.org/10.1016/j.foodchem.2016.11.085.
4. Román-Pizarro V, Fernández-Romero JM, Gómez-Hens A. (2014) Fluorometric Determination of Alkaline Phosphatase Activity in Food Using Magnetoliposomes as On-flow Microcontainer Devices. J. Agric. Food Chem. 62: 1819–1825. https://doi.org/10.1021/jf5004804.
5. Román-Pizarro V, Ramírez-Gutiérrez M, Gómez-Hens A. (2020) Fernández-Romero JM. Usefulness of magnetically controlled MNPs-enzymes microreactors for the fluorimetric determination of total cholesterol in serum. Talanta. 208: (20426). doi: 10.1016/j.talanta.2019.120426.
6. Kouassi GK, Irudayaraj J, McCarty G. (2005) Examination of cholesterol oxidase attachment to magnetic nanoparticles. Nanobiotechnology. 3 (2005) 1–9. doi:10.1186/1477-3155-3-1.
7. Écija-Arenas Á, Román-Pizarro V, Fernández-Romero JM, Gómez-Hens A (2016) Separation and purification of hydrophobic magnetite-gold hybrid nanoparticles by multiphase density gradient centrifugation. Microchimica Acta 183:2005–2012. https://doi.org/10.1007/s00604-016-1838-z
